# The Covid-19 Pandemics: why Intersectionality Matters

**DOI:** 10.3389/fsoc.2021.642662

**Published:** 2021-03-26

**Authors:** Lara Maestripieri

**Affiliations:** Department of Architecture and Urban Studies, Laboratory of Social Policies, Politecnico di Milano, Milan, Italy

**Keywords:** COVID-19, intersectionality, social distancing measures, structural inequality, gender, age, class, ethnic background

## Abstract

Covid-19 has been a disrupting event in contemporary social life but is far from being a great equaliser. Preliminary studies have put in evidence how different social groups faced a differentiated risk of contagion and coped differently with the various consequences of the emergency. Evidence shows how minorities and migrants face disproportionally higher risks of contagion than the white upper and middle class, and how vulnerable communities are more exposed to deaths and the rapid spread of the virus. At the same time, societies are coping with social distancing measures and their disruptive social and economic consequences, which have a more significant impact on the most vulnerable segments of societies: women, children, low-income classes and ethnic minorities. This article argues that an intersectional framework allows an understanding of what is occurring in the current pandemic, both in terms of its social determinants and social consequences. To open the black box of inequality, intersectional scholars analyze the intersections of multiple structures of inequalities (such as gender, age, class, ethnicity), which have a multiplying effect when disadvantaged positions intersect in the same individual. Covid-19 is a clear example of an intersectional phenomenon: the impact of individual and community exposure to Covid-19 is the results of multiple and interrelating structures of inequality. Up to now, research in social sciences has underestimated the role of intersectionality in analyzing the social and economic consequences of this pandemic.

## Introduction

Covid-19 is not a great equaliser (Alfani, 2020; Berkhout and Richardson, 2020; Fisher et al., 2020; Galasso, 2020; Lokot and Bathia, 2020). Like other more recent pandemics such as the Spanish flu in 1918 or HIV in the ’80s, the virus spread more easily among the vulnerable population. Furthermore, the economic consequences of social distancing measures are leading to an increase of inequalities compared to pre-pandemic times (Alfani, 2020; Haase, 2020; Montenovo et al., 2020; Ryan and El Ayadi, 2020) and interact with pre-existing inequalities along dimensions such as gender, age, socio-economic conditions, geography (Eaves and Al-Hindi, 2020). However, the current debate on the effects of Covid-19 has not sufficiently considered how the intersections between existing structures of inequalities affect the impact of Covid-19 on societies. In this paper, I argue that these studies have forgotten the relevance that possible intersections might have in determining the social effects of this pandemic.

The relationship between Covid-19 and the inequality structure occurs along two dimensions: how current inequality structures affect the spread of the infection and how its containment measures affect the existing systems of inequality (Holst et al., 2020). The debate about the social determinants of health is long-standing in sociology: it focuses on how education, socio-economic conditions and the environment in which people live affect their health (Scambler, 2019; Burton et al., 2020; Joseph, 2020). Covid-19 is a social disease (Trout and Kleinman, 2020): the likelihood of getting infected is influenced by economic inequalities. The virus is more likely to hit harder among those who have a concurrent illness, live in over-crowded housing or lack regular access to health services (Burton et al., 2020; Holst et al., 2020; Horton, 2020). Furthermore, Covid-19 distribution is also correlated with the vulnerability of the communities (Fisher et al., 2020; Hatef et al., 2020). When ethnically segregated, the spread of Covid-19 in a community is greater (Joseph, 2020). In the debate about social determinants of health, the need for an intersectional approach has long been recognized (Lopez and Gadsen, 2016).

But Covid-19 is not only a medical issue but a “social disaster” as well (Connell, 2020). The social distancing necessary to avoid the spread of the virus determines its social impact. These measures affect societies in three manners: 1). exposing existing vulnerabilities, 2). reinforcing current inequalities, 3). amplifying social differences in the future because of scarring effects (Haase, 2020). Thus, the scope of this review is to argue that an intersectional analytical framework is necessary to grasp the full social consequences of Covid-19.

## Exploring the Social Impact of Covid-19 Pandemics Under an Intersectional Perspective

The unprecedented measures taken for coping with Covid-19 have disrupted the labor market, with most recent ILO estimations indicating that 93% of the world’s workers experienced some sort of workplace closures. Shuts down have implied a relevant loss in working hours (8.8% compared to 2019), growing inactivity and unemployment rates, with a decline of 8.3% in labor income globally (source: ILO Monitor, seventh Edition, January 2021). The following sections review the empirical evidence emerging from on-going research conducted worldwide on the social consequences of Covid-19 social distancing measures.

### Shutdown Sectors

Measures of social distancing caused the closures of non-essential working activities with consequent massive layoffs and furloughs. Activities involving frequent interpersonal contacts suffered more prolonged closures and could not easily turn to telework. Given its characteristics, the crisis has been defined a “pink recession” as it affected especially sectors in which women are the majority of employees, with gendered effects in terms of job loss and reduction of working hours (Matthewman and Huppatz, 2020).

Adams-Prassl et al. (2020) shows that sectors such as arts and entertainment, education, food, accommodation and retail suffered in all countries considered in their study (United Kingdom, United States and Germany). The same occurred in Australia (Churchill, 2020) and Italy (Galasso, 2020). These are sectors in which women are over-represented, leading to a higher impact on their individual income (Alon et al., 2020). Blundell et al. (2020) demonstrated that in United Kingdom women, young and low-paid workers are those that suffered the most from the lockdown. In Italy, Galasso (2020) observed adverse effects on blue collars, low-educated workers and low-income services workers as well. Social distancing implied more significant job losses among ethnic minorities in the United States, especially Hispanics (Béland et al., 2020; Montenovo et al., 2020). Layoffs in the United States exacerbated pre-existing forms of parental status and gender inequality in employment, with a consistent fatherhood premium (Dias et al., 2020). Thus, mothers more than father were more likely to exit the labor force during the pandemics and, significantly, mothers of the youngest children reduced their working hours five times more than fathers (Collins et al., 2020). The 80% of job loss in Europe occurred among temporary workers, with youth employment significantly affected; the sharpest decrease occurred in elementary occupations (−10%) and sales (−8%) (Eurostat, 2020). Blundell et al. (2020) also observed a more substantial reduction in earnings for low-income workers and self-employed workers. In the global South, informal work has been significantly affected by job loss, affecting disproportionally the least well-off (Fisher et al., 2020). As such, the shutdown affected mostly vulnerable and disadvantaged workers: in general, families that were already in a condition of economic insecurity and working precariousness suffered the most from the shutdowns.

In conclusion, the more fragile workers were more likely to stop working and to suffer immediate income loss from the social distancing measures that affected mostly low-skilled services sectors (Blundell et al., 2020; Galasso, 2020). The variance in job losses in terms of demographic characteristics depends on the sorting between different sectors (Montenovo et al., 2020): the affected sectors showed a predominance of youth, minorities, women and low-skilled workers, usually employed with temporary contracts or in precarious working conditions, including solo self-employment.

### Key workers vs. Teleworking

Even during stricter restrictions, the majority of workers continued their working activities. In a study from Eurofond (2020), about half of the labor force worked at least once from home during the first months of the Covid-19 pandemics. However, working conditions were highly differentiated between those who could reorganize their work entirely at home and those who had to continue working in their usual workplace. Class divides clearly marked this separation.

The expansion of teleworking following the COVID-19 outbreak is strongly skewed toward high-paid and high-skilled white-collar employment (European Commission, 2020; Sostero et al., 2020). Galasso and Foucalt (2020), analyzing data from surveys covering several 12 OECD countries, demonstrated that college graduates and white collars were overwhelmingly working from home. Apart from belonging to the most privileged strata of labor markets, those who could telework were less likely to lose their jobs during pandemics (Adams-Prassl et al., 2020; Montenovo et al., 2020) and to suffer from their economic consequences (Béland et al., 2020). Teleworking sectors are those in which gender divides are lower. Still, women were more likely to reduce their working hours when teleworking due to the increasing burden of unpaid work with lockdown schools and the lack of help from primary networks following self-isolation (Hipp and Bünning, 2020).

Conversely, key workers belong to essential sectors such as health care, supermarkets and cleaning, and could rarely access teleworking. They are usually poorly paid with less welfare protection than other categories (Horton, 2020). Although they were not exposed to unemployment, key workers faced a higher risk of Covid-19 exposure (Montenovo et al., 2020), with consequent high proportions of contagion and deaths among them (van Barneveld et al., 2020). Essential sectors are disproportionally populated by low-skilled women and ethnic minorities, usually employed with precarious working conditions and low pay. More than 50% of those who continue working in place despite restrictions belong to the lowest educated groups in the United Kingdom (Blundell et al., 2020). Despite their essential role and the general appreciation that healthcare workers received during the pandemics, frontline workers could not capitalize on the pandemics for improving their job conditions or increasing its monetary value.

In conclusion, the capacity of teleworking reinforces pre-existing labor market inequalities. High-skilled and high-paid workers were more likely to preserve their jobs and to work safely through lockdowns, avoiding income loss. On the contrary, key frontline workers, mostly active in service and care jobs, are disproportionally low paid and more exposed to contagion. Sorting among the two groups of workers depends on education and earnings, with the most shielded group belonging to the highest classes.

### School Closures

After decades of externalization, pandemics have increased the internalization of care for dependent family members, especially children (Hupkau and Pentrongolo, 2020). School closures implied a deeper involvement of families, both in care and education; and many couples have to concentrate work and home schooling in the same (domestic) space (Craig, 2020). Social distancing also precluded support from primary networks, such as friends, neighbors or grandparents (Blaskó et al., 2020). The care burden was particularly heavy for single parents (van Barneveld et al., 2020). Not surprisingly, most of these additional care hours have been carried out by women.

School closures affected children and youth disproportionally, with a systematic and more profound impact on young from low socio-economic backgrounds (ILO, 2020). The impossibility to go to school has meant the incapacity to secure food, health, access to services for the lowest-income households (Blundell et al., 2020; Fisher et al., 2020), and widened educational inequalities among children of different socio-economic backgrounds (Reimer et al., 2020). Increase in children’s inequality occurred primarily when students were taught online (Reimer et al., 2020). In fact, digital inequalities strongly affected the capacity of families to cope with online teaching (Auriemma and Iannaccone, 2020; Puckett and Rafalow, 2020): families with lower socio-economic backgrounds had more difficulties in buying adequate instruments to support home schooling or to have sufficient space for study at home (Auriemma and Iannaccone, 2020; Blundell et al., 2020; Reimer et al., 2020). Better-off families are more likely to have both parents accessing teleworking, increasing the family’s capacity to cope with home schooling thanks to a more equal distribution of unpaid care (Craig, 2020; Del Boca et al., 2020; Hipp and Bünning, 2020). They have better houses with wider spaces and more capacity to face unexpected expenses, such as the devices needed for home schooling (Blundell et al., 2020).

Furthermore, data from live surveys in Italy (Del Boca et al., 2020), Spain (Bonal and González, 2020), Germany, Ireland and United Kingdom (Dietrich et al., 2020) show that not all the parents looked after children in the same way: mothers holding a degree and their partner spent more time on children’s education, even controlling for working arrangements. All preliminary evidence shows that schooling disruptions are likely to affect students’ cognitive development and educational attainment with 65% of young reporting lesser learning outcomes since pandemics (ILO 2020), but with differentiated effects depending on classes. Educational inequalities are more pronounced in the Global South: families with children accessing private education have been further advantaged since public schools could not provide even basic online teaching (Fisher et al., 2020).

In conclusion, the social consequences of school closures again affected the most vulnerable populations: children and young, especially if they belong to low socio-economic backgrounds or live in the Global South, might experience persisting scarring effects that might compromise their educational attainment in the future. At the same time, families were particularly stressed, with additional care burden distributed unevenly among genders.

### Home Confinement

Self-isolation imposed generally on the population implied a renovated relevance of housing in terms of accessing sufficient and secure space. Houses have suddenly become the space in which people work, study and spend their free time with the consequences of exacerbating inequalities between those who can access sufficient space for all family members and those who cannot (Auriemma and Iannaccone, 2020). Socially excluded populations, such as homeless, refugees or those who have fragile access to housing have been significantly affected by home confinement for their chronic lack of secure access to housing. Especially for undocumented migrants, their undeclared conditions implied a higher likelihood of losing their jobs when home confinement took place. Usually, they were excluded from income support schemes or housing protection measures, leaving them particularly exposed to insecurity and poverty (Guadagno, 2020).

Furthermore, home confinement and school closure have also implied an increase of gender-based violence, since women and children were locked up with their persecutors without the capacity to evade or to ask for help (Evans et al., 2020; Fisher et al., 2020). Data from the US showed an increase of up to 30% of reports on intimate violence (Eaves and Al-Hindi, 2020), a trend confirmed in China (Sacco et al., 2020). In other countries, the number of reports dramatically fell, as happened in Italy (Sacco et al., 2020), because of the impossibility for women to ask for help outside the family. Additionally, financial insecurity might force victims to stay with their abuser (OECD, 2020). Domestic violence is a phenomenon that cuts across all levels of income, education and occupation; however, empirical evidence on the Covid-19 effects on domestic violence are too preliminary to argue if they tend to concentrate among families of lower socio-economic background.

In conclusion, the adverse effects of home confinement were not evenly distributed across social groups. The preliminary empirical evidence shows the higher vulnerability of women and children, and socially excluded populations compared to the better-off families, both in terms of access to adequate housing and safe environment for study and living. Domestic violence has been on the rise for the incapacity of women to escape their persecutors during home confinement measures.

## The Intersectional Pandemic Effects

The intersectional theory has gained increased success over recent years in women’s studies for its capacity to go beyond a simple binary approach to gender inequalities. In fact, intersectionality considers what occurs when multiple axes of inequalities enter in relation, so that gender should always be seen in interactive and complex relations with other factors, such as ethnicity, class, age, etc. (Berkhout and Richardson, 2020; Eaves and Al-Hindi, 2020; Ryan and El Ayadi, 2020). Under this perspective, these structures of inequalities are «crosscutting and mutually reinforcing systems of domination and subordination», which «may construct multiple, uneven and contradictory social patterns» (Anthias 2005, 36–37). They are interrelated but different systems of inequality. Thus, people hold positions that may be conflicting with each other—such as for black middle-class women, who are privileged for their belonging to the middle class but discriminated for their being women and blacks.

As seen in the previous section (§ *Exploring the Social Impact of Covid-19 Pandemics Under an Intersectional Perspective* Section), pandemics’ direct and indirect social consequences impacted differentially on individuals belonging to different social groups. Each of the phenomenon considered (the closure of non-essential sectors, teleworkers and key workers, school closures and home confinement) had an impact differentiated by gender, class, age, ethnic background (etc.), while these structures of inequalities were mutually reinforcing each other at the intersections. For example, we can consider the case of a single mother working in an essential sector coping with the care and education of her children in the total absence of help from public services and primary networks. Her vulnerability does not merely emerge from the fact that she is female but from the contemporaneous presence of other disadvantaged conditions (being a single mother and a key worker) that magnify her vulnerability facing an adverse event (the social distancing due to pandemics).

Each social group we have identified as particularly vulnerable to the pandemic (women, young, children, ethnic minorities) is not a uniform group (Berkhout and Richardson, 2020): women differ among each other for their socio-economic conditions, if they have a partner that can support the increased burden of unpaid work determined by school closure, if they belong to vulnerable communities or have access to adequate housing. And this happens for all the other groups considered: data showed that almost three quarters of youth suffered from an interruption in their education, but for those belonging to low socio-economic classes this situation will probably imply a scarring effect in future educational attainment and, consequently, in their future capacity to secure their living on the labor market (ILO, 2020).

**FIGURE 1:**
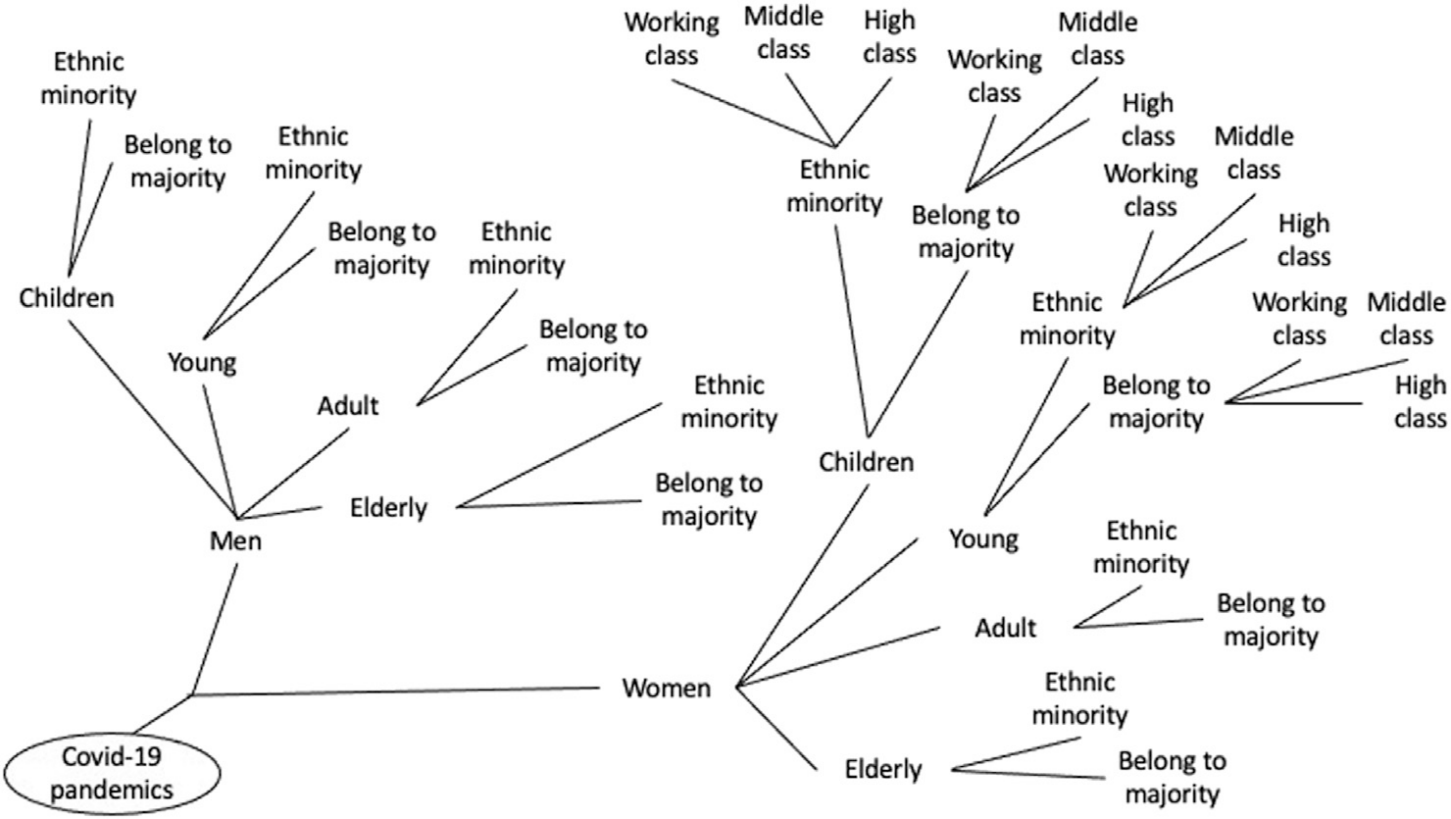
The figure represents in a simplified way the logical reasoning behind applying an intersectional approach to a social phenomenon, such as Covid-19.

Focusing on only one axis of inequality at a time impedes grasping the real reach of the pandemic effect and its unequal distribution across social groups in society. Applying an intersectional approach to Covid-19 implies using a fractal analytical approach to the complexity of reality, as shown in Figure 1: independently of which axis of inequality we consider as first, the inclusion of the others generates a tree of multiple inequalities in which the focus lies at the intersections, not at the single analytical dimension. In each intersection, the experience of Covid-19 and the relative vulnerability changes.

Measures of containment did not equally affect economic sectors, nor countries: each government decided which measure to implement, in certain cases lengthy interruptions in school provisions and longer duration of home confinement, while in others only lighter restrictions in the activities considered at highest risk of interpersonal contacts. The social and economic effects of the pandemics depended not only on the individual characteristics of the person but on the type of policy response to it, with certain countries—such as Southern Europe—more exposed than others (Moreira et al., 2020). As Wenham et al. (2020) reported, only 16 countries have enforced social protection measures that make explicit reference to women, overlooking the fact that the worst economic consequences of this crisis are likely to affect women. Quite rarely women or ethnic minorities were included in the political and technical committees that managed the emergency, and parliament’s voicing capacity was significantly undermined by the need to make rapid decisions (Blaskó et al., 2020; OECD, 2020; United Nations, 2020). The lack of an intersectional lens in assuming decisions to fight the emergency might drive away governments in their capacity to tackle the social consequences of Covid-19 effectively.

Future research with an intersectional perspective is required to understand the complexity of the current situation and tailor policy responses to cope with this complexity. The empirical evidence provided in this article has confirmed the stances of those authors that call for intersectionality in analyzing the effects of Covid-19 (Bowleg, 2020; Eaves and Al-Hindi, 2020; Ryan and El Ayadi, 2020; Wenham et al., 2020). Up to now, data have been just collected without sufficiently considering how the different structures of inequality intersect. Using an intersectional approach underlines the difference in the impact of pandemics between individuals and social groups, and helps in designing policy responses that mitigate, instead of increase, the potential unequal effect of this pandemic.
